# Enhancing molecular property prediction with auxiliary learning and task-specific adaptation

**DOI:** 10.1186/s13321-024-00880-7

**Published:** 2024-07-24

**Authors:** Vishal Dey, Xia Ning

**Affiliations:** 1 Department of Computer Science and Engineering, The Ohio State University, Columbus, 43210 OH USA; 2 Department of Biomedical Informatics, The Ohio State University, Columbus, 43210 OH USA; 3 Translational Data Analytics Institute, The Ohio State University, Columbus, 43210 OH USA

**Keywords:** Pretraining, Graph neural networks, Auxiliary learning, Task adaptation, Molecular property prediction, Drug discovery

## Abstract

Pretrained Graph Neural Networks have been widely adopted for various molecular property prediction tasks. Despite their ability to encode structural and relational features of molecules, traditional fine-tuning of such pretrained GNNs on the target task can lead to poor generalization. To address this, we explore the adaptation of pretrained GNNs to the target task by jointly training them with multiple auxiliary tasks. This could enable the GNNs to learn both general and task-specific features, which may benefit the target task. However, a major challenge is to determine the relatedness of auxiliary tasks with the target task. To address this, we investigate multiple strategies to measure the relevance of auxiliary tasks and integrate such tasks by adaptively combining task gradients or by learning task weights via bi-level optimization. Additionally, we propose a novel gradient surgery-based approach, Rotation of Conflicting Gradients ($\mathop {\texttt{RCGrad}}\limits$), that learns to align conflicting auxiliary task gradients through rotation. Our experiments with state-of-the-art pretrained GNNs demonstrate the efficacy of our proposed methods, with improvements of up to 7.7% over fine-tuning. This suggests that incorporating auxiliary tasks along with target task fine-tuning can be an effective way to improve the generalizability of pretrained GNNs for molecular property prediction.

**Scientific contribution**

We introduce a novel framework for adapting pretrained GNNs to molecular tasks using auxiliary learning to address the critical issue of negative transfer. Leveraging novel gradient surgery techniques such as $\mathop {\texttt{RCGrad}}\limits$, the proposed adaptation framework represents a significant departure from the dominant pretraining fine-tuning approach for molecular GNNs. Our contributions are significant for drug discovery research, especially for tasks with limited data, filling a notable gap in the efficient adaptation of pretrained models for molecular GNNs.

## Introduction

Accurate prediction of molecular properties is pivotal in drug discovery [39], as it accelerates the identification of potential molecules with desired properties. Developing computational models for property prediction relies on learning effective representations of molecules [5]. In this regard, Graph Neural Networks (GNNs) have shown impressive results in learning effective representations for molecular property prediction tasks [11, 12, 37]. Inspired by the paradigm of pretraining followed by fine-tuning, widely recognized for its impact in natural language understanding [27, 38], molecular GNNs are often pretrained [17] on a large corpus of molecules. Such a corpus might encompass irrelevant data for the target property prediction task. This can lead the GNNs to learn features that do not benefit the target task. Consequently, pretrained GNNs are fine-tuned with the target task to encode task-specific features. However, vanilla fine-tuning can potentially lead to poor generalization, particularly when dealing with diverse downstream tasks, limited data, and the need to generalize across varying scaffolds [40].

To improve generalization, auxiliary learning has recently garnered attention [8, 20, 21]. Auxiliary learning leverages informative signals from self-supervised tasks on unlabeled data, to improve the performance of the target tasks. However, its application in the context of molecular graphs, specifically for molecular property prediction, remains largely unexplored. Following this line of work, in this paper, we explore how to adapt pretrained molecular GNNs by combining widely-used self-supervised tasks with the target task using respective task-specific data (with self-supervised and target task labels). However, a critical challenge in such an adaptation is caused by negative transfer [29], where auxiliary tasks might impede rather than aid the target task [9, 30].

To address this challenge, we develop novel gradient surgery-based adaptation strategies, referred to as Rotation of Conflicting Gradients ($\mathop {\texttt{RCGrad}}\limits$) and Bi-level Optimization with Gradient Rotation ($\mathop {\texttt{BLO}\text {+}\texttt{RCGrad}}\limits$). Such strategies mitigate negative transfer from auxiliary tasks by learning to align conflicting gradients. Overall, our adaptation strategies improved the target task performance by as much as 7.7% over vanilla fine-tuning. Moreover, our findings indicate that the developed adaptation strategies are particularly effective in tasks with limited labeled data, which is a common challenge in molecular property prediction tasks. Our comprehensive investigation of multiple adaptation strategies for pretrained molecular GNNs represents a notable contribution in addressing the limited benefit of pretrained GNNs [34], and in improving generalizability across a diverse set of downstream tasks with limited data.

## Related work

### Pretraining and fine-tuning GNNs

Pretraining followed by fine-tuning is widely used to leverage knowledge gained from related tasks and to improve model generalization. Typically, it involves training a model on large-scale data with self-supervised or supervised tasks, and then fine-tuning it on a small-scale labeled data. Following the success of pretraining and fine-tuning paradigm in various domains [10, 23], researchers have extended it to molecular GNNs [17, 18, 22, 37]. In this regard, researchers have designed a number of self-supervised tasks as pretraining tasks that focus on capturing diverse chemical rules, connectivities, and patterns at varying granularities: on node, subgraph and graph levels [42]. Although pretrained GNNs showed promise in capturing diverse chemical knowledge, the challenge lies in effectively extracting this knowledge relevant to the target task, which is often non-trivial through vanilla fine-tuning. Specifically, such fine-tuning often leads to overfitting [41]. Contrary to the observations in domains such as natural language processing (NLP) and computer vision, where pretrained models consistently yield substantial improvements, pretrained GNNs do not exhibit such improvement [34].

This could be due to a notable research gap in determining what self-supervised molecular tasks can better benefit the downstream target tasks. In fact, prior studies in pretraining molecular GNNs mostly leverage one or two self-supervised task(s), thereby resulting in a plethora of multiple pretrained GNNs. Interestingly, such pretrained GNNs capture different knowledge [36] and excel in different downstream molecular property prediction tasks [34]. Additionally, Sun et al. [34] recently demonstrated that self-supervised graph pretraining does not consistently/significantly outperform non-pretraining methods across various settings. Overall, although pretrained GNNs hold promise for molecular property prediction, their benefit over non-pretrained models seems limited. To address this, some recent attempts [41, 46] to fine-tune pretrained GNNs have largely relied on existing ideas like regularization [43] or update constraints [16] during fine-tuning. In contrast, our proposed approaches leverage auxiliary tasks to learn generalizable knowledge and prevent overfitting to the training set.

### Knowledge transfer with auxiliary learning

Knowledge transfer through auxiliary learning has demonstrated its effectiveness across a spectrum of domains [19, 26, 35]. This paradigm, distinct from multi-task learning, aims to optimize the target task’s performance while leveraging auxiliary tasks to bolster generalization [32]. Prior research in other domains has developed multiple methods to automatically learn task weights, such as using gradient similarity [6, 9], using parameterized auxiliary network [8, 25], using bi-level optimization and implicit differentiation [2, 25], minimizing distances between task embeddings [3], or from the perspective of Nash equilibrium [31]. However, the application of auxiliary learning for adapting molecular GNNs to target tasks, particularly in the context of molecular property prediction, remains an under-explored area. In this study, we adopt and explore gradient similarity, gradient scaling, and bi-level optimization strategies.

## Preliminaries

Motivated by the success of continued pretraining and task-specific adaptation in pretrained Large Language Models (LLMs) [7, 13, 44], we investigate adaptation of off-the-shelf pretrained molecular GNNs to target molecular property prediction tasks. Via such an adaptation, we aim to leverage existing self-supervised (SSL) tasks designed for molecular GNNs and transfer learned knowledge from such tasks to the target task. We employ the existing SSL tasks typically used in molecular pretraining such as masked atom prediction (AM), context prediction (CP) [17], edge prediction (EP) [14], graph infomax (IG) [33], and motif prediction (MP) [28]. (detailed in B.1). We refer to these tasks as auxiliary tasks. Intuitively, these auxiliary tasks can potentially capture diverse chemical semantics and rich structural patterns at varying granularities. By utilizing SSL objectives on target task-specific data, auxiliary tasks augment the pretrained GNNs with richer representations. Such representations, in turn, can improve the generalizability of the target property prediction task. Henceforth, the term “GNN” refers to an off-the-shelf pretrained molecular GNN.

**Fig. 1:**
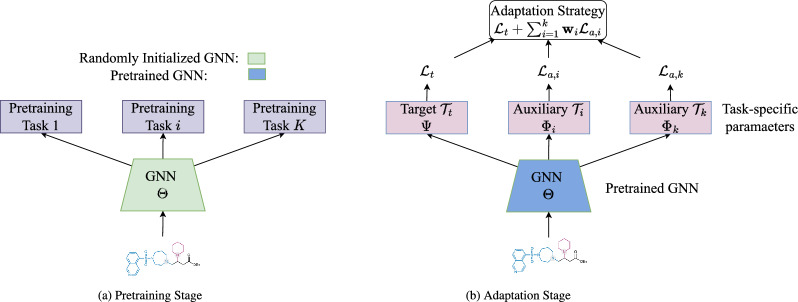
Off-the-shelf available pretrained GNNs are transferred for target task-specific adaptation

Figure 1 presents an overview of the adaptation setup. Formally, we adapt a GNN with parameters $\Theta$ to optimize the performance on the target task $\mathop {\mathcal {T}_{t}}\limits$. We achieve this by jointly training $\mathop {\mathcal {T}_{t}}\limits$ with auxiliary tasks $\{\mathop {\mathcal {T}_{a,i}}\limits \}^k_{i=1}$ through solving the following optimization problem:

1 $$\begin{aligned} \min _{ {\Theta ,\Psi ,\Phi}_{i\in \{1..k\}} } {\mathcal{L}_{t}} + \sum _{i=1}^k {{\textbf{w}}_i} {\mathcal {L}_{a,i}} , \end{aligned}$$

where $\mathop {\mathcal {L}_{t}}\limits$ and $\mathop {\mathcal {L}_{a,i}}\limits$ denote the target task loss and *i*-th auxiliary task loss, respectively, $\Psi$ and $\Phi _{i\in \{1,...,k\}}$ denotes task-specific learnable parameters for the target and *i*-th auxiliary task, respectively, and $\textbf{w}$ is the weight indicating the influence of the auxiliary tasks on the target task. Through the above optimization, all the parameters are simultaneously updated in an end-to-end manner. Note that the above optimization does not optimize $\textbf{w}$– we will introduce an approach that can additionally learn $\textbf{w}$ in Section ''Bi-Level Optimization''. In fact, the key to effective adaptation lies in accurately determining $\textbf{w}$, such that the combined task gradients can backpropagate relevant training signals to the shared GNN as follows:

$$\begin{aligned}\Theta ^{(t+1)} := \Theta ^{(t)} - \alpha \left( \mathop {\textbf{g}_{t}}\limits + \sum \nolimits _{i=1}^k \textbf{w} _i \mathop {\textbf{g}_{a,i}}\limits \right) ,\end{aligned}$$

where $\mathop {\textbf{g}_{t}}\limits = \mathop {\nabla _{{\Theta }}}\limits \mathop {\mathcal {L}_{t}}\limits$, and $\mathop {\textbf{g}_{a,i}}\limits = \mathop {\nabla _{{\Theta }}}\limits \mathop {\mathcal {L}_{a,i}}\limits$ denote the gradients updating $\Theta$ from the target and *i*-th auxiliary task, respectively, and $\alpha$ denotes the learning rate. Our proposed adaptation strategies focus on learning such $\textbf{w}$ in an end-to-end manner, to dynamically combine task gradients during each update. These strategies contrast with those using fixed weights or conducting expensive grid-search to explore all possible $\textbf{w}$.

### Gradient cosine similarity ($\mathop {\texttt{GCS}}\limits$)

The first strategy to meaningfully combine task gradients is based on gradient cosine similarity ($\mathop {\texttt{GCS}}\limits$) [9]. Intuitively, $\mathop {\texttt{GCS}}\limits$ measures the alignment between task gradients during training, providing insights into the relatedness of auxiliary tasks with the target task. A high $\mathop {\texttt{GCS}}\limits$ indicates that the auxiliary tasks provide complementary information, and thus, can benefit the target task. Conversely, low $\mathop {\texttt{GCS}}\limits$ indicates potential orthogonality or even conflict between tasks. Thus, $\mathop {\texttt{GCS}}\limits$ can naturally quantify the relatedness of auxiliary tasks with the target task over the course of training. We compute $\mathop {\texttt{GCS}}\limits$ and update $\Theta$ as:

$$\begin{aligned}\Theta ^{(t+1)} := \Theta ^{(t)} - \alpha \left( \mathop {\textbf{g}_{t}}\limits + \sum \nolimits _{i=1}^k \max \left( 0, \cos \left( \mathop {\textbf{g}_{t}}\limits , \mathop {\textbf{g}_{a,i}}\limits )\right) \mathop {\textbf{g}_{a,i}}\limits \right) \right) ,\end{aligned}$$

where, $\max$ operator takes the maximum out of the two values, thereby, dropping the tasks with conflicting gradients (i.e., with negative $\mathop {\texttt{GCS}}\limits$).

**Fig. 2:**
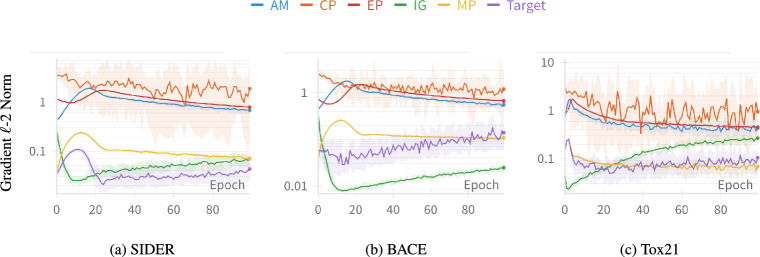
Large variations of scales among task gradients are observed when ${\mathop {\mathtt {Sup\text {-}CP}}\limits }$ is adapted with all auxiliary tasks using $\mathop {\texttt{MTL}}\limits$

### Gradient scaling ($\mathop {\texttt{GNS}}\limits$)

We also adopt a simpler strategy of gradient scaling [15] to adjust the influence of auxiliary tasks with respect to the target task. Our preliminary experiments as presented in Figure 2 revealed significant differences in the scales of the task gradient norms, and thus requiring careful adjustments. This is because if the gradient of an auxiliary task is much larger than that of the target task, $\Theta$ updates will be most dominated by such auxiliary tasks, thereby potentially resulting in worse target performance. On the other hand, if the gradient of an auxiliary task is relatively small, the training signals from such auxiliary tasks will be too weak to encode any relevant features in $\Theta$. Thus, following [4, 15], we use a simple gradient scaling to dynamically adjust the influence of auxiliary tasks during updates of $\Theta$ as follows:

2 $$\begin{aligned} \!\!\!\!\Theta ^{(t+1)} := \Theta ^{(t)} - \alpha \left( \mathop {\textbf{g}_{t}}\limits + \sum _{i=1}^k \max \left( 1, \frac{||\mathop {\textbf{g}_{t}}\limits ||}{||\mathop {\textbf{g}_{a,i}}\limits ||}\right) \mathop {\textbf{g}_{a,i}}\limits \right) , \end{aligned}$$

where $||\cdot ||$ denotes the $\ell$-2 norm.

## Methods

**Fig. 3:**
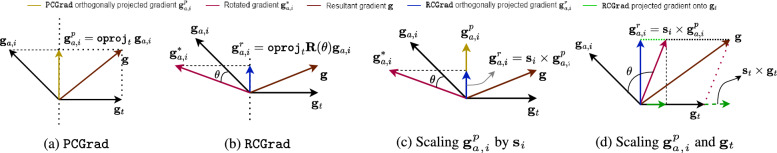
**a** $\mathop {\texttt{PCGrad}}\limits$ projects conflicting gradient $\mathop {\textbf{g}_{a,i}}\limits$ onto the normal plane of $\mathop {\textbf{g}_{t}}\limits$. **b** $\mathop {\texttt{RCGrad}}\limits$ applies a rotation to $\mathop {\textbf{g}_{a,i}}\limits$, followed by projection. **c** Rotation followed by orthogonal projection is equivalent to scaling $\mathop {\textbf{g}^p_{a,i}}\limits$. **d** If the rotated gradient does not conflict with $\mathop {\textbf{g}_{t}}\limits$, the projection of the rotated gradient onto $\mathop {\textbf{g}_{t}}\limits$ is incorporated as scaling $\mathop {\textbf{g}_{t}}\limits$ by $(1+\textbf{s} _t)$

### Rotation of conflicting gradients ($\mathop {\texttt{RCGrad}}\limits$)

While both conflicting directions and magnitude differences of task gradients can lead to negative transfer, $\mathop {\texttt{GCS}}\limits$ and $\mathop {\texttt{GNS}}\limits$ focus separately on homogenizing either the direction or magnitude of gradients, rather than in a unified manner. To address these limitations, we develop Rotation of Conflicting Gradients ($\mathop {\texttt{RCGrad}}\limits$) – a novel extension of $\mathop {\texttt{PCGrad}}\limits$ [45] – that aligns gradients both in terms of direction and magnitude. $\mathop {\texttt{RCGrad}}\limits$, which builds upon $\mathop {\texttt{PCGrad}}\limits$, does not completely discard gradients conflicting with the target task, unlike $\mathop {\texttt{GCS}}\limits$. Instead, $\mathop {\texttt{RCGrad}}\limits$ only negates the component of the conflicting gradient that is completely opposite to the target task gradient. Additionally, $\mathop {\texttt{RCGrad}}\limits$ explicitly learns how much of the non-conflicting component should be incorporated for the most effective knowledge transfer. This mitigates negative transfer by not only removing the conflicting component but also by learning to incorporate a portion of the non-conflicting component.

Figure 3 demonstrates the difference between $\mathop {\texttt{PCGrad}}\limits$ and $\mathop {\texttt{RCGrad}}\limits$. Formally, $\mathop {\texttt{RCGrad}}\limits$ learns to rotate auxiliary gradient ${\textbf{g}}_{a,i}$ by angle $\theta _i$ to yield a rotated gradient ${\textbf{R}}(\theta _i)\mathop {\textbf{g}_{a,i}}\limits$, which is followed by an orthogonal projection in case of conflicts (Figure 3b). The orthogonally projected component is computed as $\mathop {\textbf{g}^r_{a,i}}\limits = \mathtt{oproj}_{t} ~{\textbf{R}}(\theta _i)\mathop {\textbf{g}_{a,i}}\limits$, where ${\textbf{R}}(\theta _i)$ is the rotation matrix parameterized by $\theta _i$, and $\texttt{oproj}_t$ is the orthogonal vector projection operator as defined in Eq. 3. Via such an operator (Figure 3a), $\mathop {\texttt{PCGrad}}\limits$ projects the conflicting auxiliary gradient $\mathop {\textbf{g}_{a,i}}\limits$ onto the normal plane of the target task’s gradient $\mathop {\textbf{g}_{t}}\limits$ to yield $\mathop {\textbf{g}^p_{a,i}}\limits$ as follows:

3 $$\begin{aligned} \mathop {\textbf{g}^p_{a,i}}\limits = \mathtt{oproj}_{t} ~\mathop {\textbf{g}_{a,i}}\limits = \mathop {\textbf{g}_{a,i}}\limits - \frac{\mathop {\textbf{g}_{a,i}}\limits \cdot \mathop {\textbf{g}_{t}}\limits }{||\mathop {\textbf{g}_{t}}\limits ||} \cdot \frac{\mathop {\textbf{g}_{t}}\limits }{||\mathop {\textbf{g}_{t}}\limits ||}, \end{aligned}$$

where $\texttt{oproj}_t$ denotes the orthogonal projection operator with respect to $\mathop {\textbf{g}_{t}}\limits$. This enables effective knowledge transfer from auxiliary tasks, even if they share some dissimilarity to the target task. However, $\mathop {\texttt{PCGrad}}\limits$ does not explicitly learn how much of the non-conflicting component should be incorporated for the most effective knowledge transfer. To address this limitation, $\mathop {\texttt{RCGrad}}\limits$ learns an appropriate rotation to be applied to the auxiliary gradient $\mathop {\textbf{g}_{a,i}}\limits$, followed by the projection of the rotated gradient. Such a learnable rotation in an end-to-end manner enables dynamic knowledge transfer from auxiliary tasks such that the target task performance can be improved.

Moreover, as shown in Figures 3c, d, the rotation followed by the projection of gradients is equivalent to applying appropriate scaling factors $\textbf{s} _i$ and $\textbf{s} _t$ on the projected gradients $\mathop {\textbf{g}^p_{a,i}}\limits$ and $\mathop {\textbf{g}_{t}}\limits$, respectively. Additionally, different from $\mathop {\texttt{PCGrad}}\limits$, $\mathop {\texttt{RCGrad}}\limits$ accounts for large differences in gradient magnitudes by adjusting the magnitudes of non-conflicting auxiliary task gradients relative to that of the target task gradient (Eq. 2). To summarize, $\Theta$ is updated as follows: $\Theta ^{(t+1)} := \Theta ^{(t)} - \alpha {\textbf{g}}$, where ${\textbf{g}} =(1+\textbf{s} _t) \times \mathop {\textbf{g}_{t}}\limits + \sum _{i=1}^k \mathop {\textbf{g}^r_{a,i}}\limits$, and

4 $$\begin{aligned} \mathop {\textbf{g}^r_{a,i}}\limits = {\left\{ \begin{array}{ll} \textbf{s} _i \times \mathop {\textbf{g}^p_{a,i}}\limits &{} \text { if } \mathop {\textbf{g}_{t}}\limits \cdot \mathop {\textbf{g}_{a,i}}\limits < 0, \\ \max \left( 1, \frac{||\mathop {\textbf{g}_{t}}\limits ||}{||\mathop {\textbf{g}_{a,i}}\limits ||}\right) \mathop {\textbf{g}_{a,i}}\limits &{} \text { otherwise}, \end{array}\right. } \end{aligned}$$

where $\mathop {\textbf{g}^p_{a,i}}\limits$ is computed via Eq. 3. Note that the set of scaling factors $\textbf{s} =\{\{\textbf{s} _i\}_{i=1}^k, \textbf{s} _t\}$ is learned in an end-to-end manner during the optimization of the combined losses from all tasks.

### Bi-level optimization ($\mathop {\texttt{BLO}}\limits$)

Unlike the previous approaches that directly manipulate task gradients, $\mathop {\texttt{BLO}}\limits$ learns task weights $\textbf{w}$ (Eq. 1) in an end-to-end manner, such that the GNN generalizes well to the target task. Note that $\mathop {\texttt{BLO}}\limits$ does not directly intervene in the gradient computation process. Instead, $\mathop {\texttt{BLO}}\limits$ learns $\textbf{w}$ that minimizes the target validation loss while ensuring that the GNN is optimized with a weighted combination of losses:

5 $$\begin{aligned} \begin{aligned} \textbf{w} ^*&= \arg \min \nolimits _{{\textbf{w}}} \mathop {\mathcal {L}_{t}}\limits ^{{(\mathop {\mathcal {A}}\limits )}}(\Theta ^*(\textbf{w})), ~~~~~~~\text {s.t.} ~~~~~~~ \Theta ^*(\textbf{w})&= \arg \min \nolimits _{{\Theta }} \mathop {\mathcal {L}_{f}}\limits (\Theta , \textbf{w}) \end{aligned} \end{aligned}$$

where, $\mathop {\mathcal {L}_{f}}\limits = \mathop {\mathcal {L}_{t}}\limits + \sum _{i=1}^k\textbf{w} _i\mathop {\mathcal {L}_{a,i}}\limits$ is the combined loss on the training set, and $\mathop {\mathcal {L}_{t}}\limits ^{{(\mathop {\mathcal {A}}\limits )}}$ is the loss on the target task computed with a held-out auxiliary dataset $\mathop {\mathcal {A}}\limits$, and $\Theta ^*(\textbf{w})$ is the best-response of $\Theta$ with current $\textbf{w}$. This formulation is a bi-level optimization problem: updating $\textbf{w}$ in the upper-level optimization requires computing $\mathop {\nabla _{{\textbf{w}}}}\limits \mathop {\mathcal {L}_{t}^{{(\mathop {\mathcal {A}}\limits )}}}\limits = \mathop {\nabla _{{\Theta }}}\limits \mathop {\mathcal {L}_{t}^{{(\mathop {\mathcal {A}}\limits )}}}\limits \cdot \mathop {\nabla _{{\textbf{w}}}}\limits \Theta ^*$, where the latter gradient requires back-propagation through the inner-level optimization of $\Theta$. Following [24], we leverage the Implicit Function Theorem (IFT) to compute $\mathop {\nabla _{{\textbf{w}}}}\limits \Theta ^* = -(\mathop {\nabla ^2_{{\Theta }}}\limits \mathop {\mathcal {L}_{f}}\limits )^{-1} \cdot \mathop {\nabla _{{\textbf{w}}}}\limits \mathop {\nabla _{{\Theta }}}\limits \mathop {\mathcal {L}_{f}}\limits$. Intuitively, IFT allows us to evaluate the $\mathop {\nabla _{{\textbf{w}}}}\limits \Theta ^*$ locally around the approximate best-response $\Theta ^*$. Using the above, we can compute the gradients $\mathop {\nabla _{{\textbf{w}}}}\limits$ $\mathop {\mathcal {L}_{t}^{{(\mathop {\mathcal {A}}\limits )}}}\limits$ as:

6 $$\begin{aligned} \begin{aligned} \mathop {\nabla _{{\textbf{w}}}}\limits \mathop {\mathcal {L}_{t}^{{(\mathop {\mathcal {A}}\limits )}}}\limits (\Theta ^*(\textbf{w}))&= \mathop {\nabla _{{\Theta }}}\limits \mathop {\mathcal {L}_{t}^{{(\mathop {\mathcal {A}}\limits )}}}\limits \cdot \mathop {\nabla _{{\textbf{w}}}}\limits \Theta ^*(\textbf{w}) \\&= - \mathop {\nabla _{{\Theta }}}\limits \mathop {\mathcal {L}_{t}^{{(\mathop {\mathcal {A}}\limits )}}}\limits \cdot (\mathop {\nabla ^2_{{\Theta }}}\limits \mathop {\mathcal {L}_{f}}\limits )^{-1} \cdot \mathop {\nabla _{{\textbf{w}}}}\limits \mathop {\nabla _{{\Theta }}}\limits \mathop {\mathcal {L}_{f}}\limits . \end{aligned} \end{aligned}$$

We described the entire training process in Algorithm 1 (Appendix A). To compute the Hessian inverse and vector products efficiently, we use the iterative algorithm by Lorraine et al. [24], which is summarized in Algorithm 2 (Appendix A). Intuitively, it uses a Neumann series expansion to approximate the Hessian inverse with unrolling differentiation for *M* steps around locally approximate best-response $\Theta ^*$. Following [25], in practice, we don’t train $\Theta$ till convergence (i.e., $\Theta ^*$ such that $\mathop {\nabla _{{\Theta }}}\limits \mathop {\mathcal {L}_{f}}\limits = 0$). Instead, we approximate $\Theta ^*$ by simultaneously training both $\Theta$ and $\textbf{w}$, and alternately optimizing $\textbf{w}$ for every *r* updates of $\Theta$. We refer the readers to [24] for theoretical considerations on approximations and convergence. Note that we use 20% of the training set as $\mathop {\mathcal {A}}\limits$ instead of using the validation set to avoid data leakage and unfair comparison with baselines. Optimizing $\textbf{w}$ on a held-out $\mathop {\mathcal {A}}\limits$ rather than on the training set aligns with the goal of improving target task generalizability.

### $\mathop {\texttt{BLO}}\limits$ with gradient rotation ($\mathop {\texttt{BLO}\text {+}\texttt{RCGrad}}\limits$)

In the previous sections, we discussed $\mathop {\texttt{RCGrad}}\limits$, which learns to project and scale conflicting gradients using $\textbf{s}$, and $\mathop {\texttt{BLO}}\limits$, which learns task weights $\textbf{w}$ but does not explicitly handle gradient conflicts. In this section, we introduce a novel approach $\mathop {\texttt{BLO}\text {+}\texttt{RCGrad}}\limits$ that combines the strengths of both $\mathop {\texttt{RCGrad}}\limits$ and $\mathop {\texttt{BLO}}\limits$. Instead of learning the scaling factors $\textbf{s}$ by minimizing the combined loss on the training split as in $\mathop {\texttt{RCGrad}}\limits$, $\mathop {\texttt{BLO}\text {+}\texttt{RCGrad}}\limits$ learns $\textbf{s}$ that minimizes the target validation loss, which is similar to the optimization of $\textbf{w}$ in $\mathop {\texttt{BLO}}\limits$. This enables learning $\textbf{s}$ that can effectively homogenize conflicting task gradients based on the generalization performance of the target task. In $\mathop {\texttt{BLO}\text {+}\texttt{RCGrad}}\limits$, the bi-level optimization is employed for learning $\textbf{s}$ not to balance task losses but to best align conflicting task gradients. This addresses the limitation of $\mathop {\texttt{BLO}}\limits$ in handling gradient conflicts by incorporating the rotational alignment strategy of $\mathop {\texttt{RCGrad}}\limits$. To summarize, $\mathop {\texttt{BLO}\text {+}\texttt{RCGrad}}\limits$ leverages the learned scaling factors $\textbf{s}$ via $\mathop {\texttt{BLO}}\limits$ (Algorithm 1) to guide the gradient surgery process introduced by $\mathop {\texttt{RCGrad}}\limits$ (Eq. 4). This dynamically controls the knowledge transfer from auxiliary tasks, ensuring that the influence of each task is optimally tuned to benefit the target task learning.

**Table 1:** Test ROC-AUC using ${\mathop {\mathcal {T}_{a}}\limits }$={AM,CP,EP,IG,MP} and ${\mathop {\mathtt {Sup\text {-}CP}}\limits }$

Method	SIDER	ClinTox	BACE	BBBP	Tox21	ToxCast	HIV	MUV
$\mathop {\texttt{FT}}\limits$	61.82 (0.53)	**71.10** (1.40)	82.86 (0.87)	67.57 (1.39)	77.05 (0.34)	66.02 (0.18)	78.70 (0.80)	80.64(0.51)
$\mathop {\texttt{GTOT}}\limits$	62.24 (0.34)	70.03 (1.58)	83.67 (1.75)	69.01 (1.95)	**77.08** (0.66)	65.45 (0.45)	**80.05** (0.57)	82.09(3.13)
$\mathop {\texttt{MTL}}\limits$	56.22 (0.82)	56.41 (3.43)	80.04 (1.48)	64.88(1.23)	74.42 (0.34)	64.53 (0.38)	76.79 (0.26)	81.68 (0.49)
$\mathop {\texttt{GCS}}\limits$	59.94 (0.53)	62.77 (2.17)	85.60 (0.63)	71.16(0.52)	74.76 (0.42)	66.05 (0.19)	76.94 (1.30)	76.65 (1.17)
$\mathop {\texttt{GNS}}\limits$	62.48 (0.56)	67.94 (1.02)	84.80 (0.34)	70.94(0.86)	76.44 (0.24)	66.19 (0.21)	78.23 (0.44)	**83.50** (1.14)
$\mathop {\texttt{PCGrad}}\limits$	62.09 (0.62)	67.60 (1.88)	84.42 (1.23)	69.14 (1.25)	76.58 (0.77)	65.81 (0.61)	77.76 (1.08)	78.62 (0.29)
$\mathop {\texttt{BLO}}\limits$	60.70 (2.37)	68.29 (3.02)	85.14 (1.23)^a^	69.80 (0.68)	76.57 (0.44)	65.80 (0.79)	79.16 (0.47)	82.19(1.40)
$\mathop {\texttt{RCGrad}}\limits$	**62.49** (0.65)	**70.07 **(1.70)^b^	**85.65** (0.60)^a^	**72.35** (1.28)^ab^	**77.26**(0.38)^b^	**66.49** (0.30)^ab^	**79.39** (0.63)	83.07 (1.26)
$\mathop {\texttt{BLO}\text {+}\texttt{RCGrad}}\limits$	**62.94** (0.66)^a^	69.59 (2.12)^b^	**86.10** (0.35)^a^	**71.81** (1.57)^a^	76.62 (0.29)	**66.38** (0.29)^a^	79.03 (1.12)	**83.92 **(1.03)

We report the mean (and standard deviation) over 10 different seeds with scaffold splitting. Best- and second best-performing models are in **bold** and bold. Tasks are presented in increasing order of size. a and b indicate statistical significance compared to the best finetuning and $\mathop {\texttt{GS}}\limits$ baselines, respectively. Statistical significance is determined based on the Wilcoxon signed rank test with p < 0.05

## Results and discussion

### Experimental setup

We perform experiments on 8 benchmark classification datasets from MoleculeNet [40]. We compare our adaptation strategies with simple baselines such as traditional fine-tuning ($\mathop {\texttt{FT}}\limits$), and vanilla multi-task learning ($\mathop {\texttt{MTL}}\limits$) that assigns equal weights to all auxiliary tasks; and a more advanced state-of-the-art regularization-based fine-tuning with optimal transport ($\mathop {\texttt{GTOT}}\limits$) [46]. Additionally, we consider other state-of-the-art gradient surgery-based methods ($\mathop {\texttt{GCS}}\limits$, $\mathop {\texttt{GNS}}\limits$, $\mathop {\texttt{PCGrad}}\limits$) as baselines. We refer to this group of baselines collectively as $\mathop {\texttt{GS}}\limits$ methods. We use the official publicly available checkpoints^1^ of two GNNs: 1) supervised_contextpred [17], denoted as ${\mathop {\mathtt {Sup\text {-}CP}}\limits }$, which is pretrained via self-supervised context prediction and supervised graph-level multi-task learning, and 2) supervised [17], denoted as $\mathop {\texttt{Sup}}\limits$, which is pretrained only via supervised graph-level multi-task learning. Using such different pretrained GNNs allows a controlled comparison to understand how different pretraining objectives (with and without self-supervised context prediction task) can influence the adaptation. Details on auxiliary tasks and datasets are presented in Table 4 and Appendix B.

### Reproducibility and implementation details

Following the prior line of research [17, 22], we use scaffold-split for the downstream target tasks, and use the same atom and bond features as in $\mathop {\texttt{GTOT}}\limits$. All experimental details for the $\mathop {\texttt{FT}}\limits$ baseline follow the $\mathop {\texttt{GTOT}}\limits$ fine-tuning setup. Specifically, we initialized a linear projection layer on top of the pretrained GNN as the target task classifier. Across all methods, both the pretrained GNN and task-specific layers are trainable. For $\mathop {\texttt{FT}}\limits$ and adaptation methods, we train the models for 100 epochs with Adam optimizer with an initial learning rate $\alpha$ of 0.001, we use a batch size of {32, 64, 256}, an embedding dimension of 300, and a dropout probability of 0.5 for the GNN module. For $\mathop {\texttt{GTOT}}\limits$ experiments, we use the optimal hyper-parameters provided for each dataset, when finetuned on ${\mathop {\mathtt {Sup\text {-}CP}}\limits }$. For $\mathop {\texttt{MTL}}\limits$ experiments, we assign equal weights to all auxiliary tasks. For $\mathop {\texttt{BLO}}\limits$ and $\mathop {\texttt{BLO}\text {+}\texttt{RCGrad}}\limits$ experiments, we use $M=3$ in Algorithm 2, update $\textbf{w}$ every $r=\{5,10,20\}$ update of $\Theta$, and use Adam optimizer with learning rate $\beta$ of 0.001 to update $\textbf{w}$. The code is available at https://github.com/vishaldeyiiest/GraphTA.

### Comparison using ${\mathop {\mathtt {Sup\text {-}CP}}\limits }$as the pretrained GNN

Table 1 presents an overall comparison when all the auxiliary tasks are used with ${\mathop {\mathtt {Sup\text {-}CP}}\limits }$as the pretrained GNN. Our proposed adaptation strategies, specifically $\mathop {\texttt{RCGrad}}\limits$ and $\mathop {\texttt{BLO}\text {+}\texttt{RCGrad}}\limits$, outperform all baselines, including other $\mathop {\texttt{GS}}\limits$-based adaptation strategies, across all datasets (except ClinTox). Specifically, compared to the best fine-tuning method, $\mathop {\texttt{GTOT}}\limits$, $\mathop {\texttt{RCGrad}}\limits$ demonstrated significant improvement of 2.4% and 4.8% in BACE and BBBP, respectively. This indicates the efficacy of our proposed rotational alignment in mitigating negative transfer and improving the generalizability of the pretrained GNN. Furthermore, $\mathop {\texttt{BLO}\text {+}\texttt{RCGrad}}\limits$ exhibits significant improvement over fine-tuning methods $\mathop {\texttt{FT}}\limits$ and $\mathop {\texttt{GTOT}}\limits$ in small-scale datasets of as much as 6.3% and 4.1%, respectively. This highlights the efficacy of bi-level optimization combined with gradient rotation in improving generalizability, especially in limited data regimes.

Additionally, $\mathop {\texttt{RCGrad}}\limits$ and $\mathop {\texttt{BLO}\text {+}\texttt{RCGrad}}\limits$ consistently outperform other gradient surgery-based ($\mathop {\texttt{GS}}\limits$) methods. Specifically, compared to $\mathop {\texttt{PCGrad}}\limits$, $\mathop {\texttt{RCGrad}}\limits$ demonstrates statistically significant improvements in ROC-AUC by 2.5%, 4.7%, 0.9% and 1.0% in ClinTox, BBBP, Tox21, and ToxCast, respectively. This improvement can be attributed to the rotation component in $\mathop {\texttt{RCGrad}}\limits$, which not only resolves gradient conflicts but also actively aligns them in a direction favorable to the target task. Moreover, our proposed methods $\mathop {\texttt{RCGrad}}\limits$ and $\mathop {\texttt{BLO}\text {+}\texttt{RCGrad}}\limits$ learn to retain a component of the conflicting task gradients, unlike $\mathop {\texttt{GCS}}\limits$ which completely discards conflicting gradients. This ensures that valuable information from auxiliary tasks is not discarded, thus facilitating more effective knowledge transfer.

Conversely, $\mathop {\texttt{BLO}}\limits$, which learns task weights without explicitly handling gradient conflicts, performs comparably or slightly worse than $\mathop {\texttt{RCGrad}}\limits$, $\mathop {\texttt{BLO}\text {+}\texttt{RCGrad}}\limits$, and other $\mathop {\texttt{GS}}\limits$-based baselines. The suboptimal performance of $\mathop {\texttt{BLO}}\limits$, especially in smaller datasets (e.g., SIDER), may be attributed to the noisy nature of task gradients, potentially leading to a poor approximation of hyper-gradients. In contrast, $\mathop {\texttt{GNS}}\limits$ is more robust to noisy gradients since it adjusts the scale of gradient magnitudes relative to the target task. Overall, our proposed methods consistently outperform all baselines on smaller datasets (except ClinTox), while achieving competitive performance on larger ones.

**Fig. 4:**
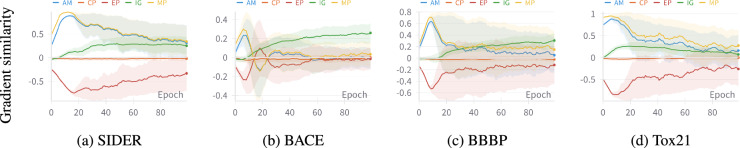
Target task gradient conflicts with EP and CP tasks. ${\mathop {\mathtt {Sup\text {-}CP}}\limits }$is adapted with all auxiliary tasks in a $\mathop {\texttt{MTL}}\limits$ setting

In contrast, $\mathop {\texttt{MTL}}\limits$, which assigns equal weights to all auxiliary tasks regardless of their relevance to the target task, results in worse performance across all downstream tasks. Compared to $\mathop {\texttt{FT}}\limits$, $\mathop {\texttt{MTL}}\limits$ exhibits deteriorations of as much as 9.1% and 20.6% in SIDER and ClinTox, respectively. This indicates that $\mathop {\texttt{MTL}}\limits$ leads to drastic negative transfer, where the auxiliary tasks hurt the performance of the target task. On the contrary, all adaptation strategies (including $\mathop {\texttt{GS}}\limits$-based baselines) perform better than $\mathop {\texttt{MTL}}\limits$ with significant improvements of up to 24.2%. Furthermore, upon analyzing gradient similarities of auxiliary tasks with the target task (Figure 4), we hypothesize that AM, IG, and MP may benefit the target task better than the other auxiliary tasks.

**Table 2:** Test ROC-AUC using ${\mathop {\mathcal {T}_{a}}\limits }$={AM,IG,MP} and ${\mathop {\mathtt {Sup\text {-}CP}}\limits }$

Method	SIDER	ClinTox	BACE	BBBP	Tox21	ToxCast	HIV	MUV
$\mathop {\texttt{FT}}\limits$	61.82 (0.53)	**71.10** (1.40)	82.86 (0.87)	67.57 (1.39)	77.05 (0.34)	66.02 (0.18)	78.70 (0.80)	80.64 (0.51)
$\mathop {\texttt{GTOT}}\limits$	62.24 (0.34)	70.03 (1.58)	83.67 (1.75)	69.01 (1.95)	**77.08** (0.66)	65.45 (0.45)	**80.05** (0.57)	82.09 (3.13)
$\mathop {\texttt{MTL}}\limits$	59.15 (1.84)	62.01 (1.87)	83.60 (0.43)	71.67 (4.44)	75.64 (0.37)	65.14 (0.21)	78.18 (1.07)	81.26 (1.90)
$\mathop {\texttt{GCS}}\limits$	62.83 (0.70)	64.62 (1.83)	84.17 (0.87)	70.49 (4.26)	**77.35** (0.20)	66.03 (0.12)	77.59 (1.24)	80.17 (3.26)
$\mathop {\texttt{GNS}}\limits$	62.62 (0.49)	63.42 (2.19)	84.29 (0.97)	71.79 (3.72)	76.50 (0.39)	66.12 (0.20)	78.25 (0.60)	82.42 (0.47)
$\mathop {\texttt{PCGrad}}\limits$	61.42 (1.69)	63.44 (2.90)	83.92 (1.23)	70.86 (4.54)	76.73 (0.89)	65.96 (0.71)	77.38 (1.10)	80.45 (2.34)
$\mathop {\texttt{BLO}}\limits$	**62.85** (0.77)	67.52 (3.27)^b^	**84.79** (0.62)	71.93 (3.19)^a^	76.88 (0.26)	**66.29** (0.26)^a^	79.21 (0.33)	81.86 (1.19)
$\mathop {\texttt{RCGrad}}\limits$	**63.12 **(0.38)^ab^	69.91 (1.22)^b^	**85.86 **(0.38)^ab^	**72.76 **(1.05)^a^^a^	76.86 (0.38)	**66.37**(0.16)^ab^	79.17 (0.32)	**82.68** (1.92)
$\mathop {\texttt{BLO}\text {+}\texttt{RCGrad}}\limits$	62.44 (0.28)	**70.99** (2.31)^b^	84.75 (0.53)	**72.03** (3.83)^a^	76.64 (0.28)	66.25 (0.22)^ab^	**79.75** (0.81)	**82.65** (3.36)

Best- and second best-performing models are in **bold** and bold. a and b indicate statistical significance compared to the best baselines based on the Wilcoxon signed rank test with p < 0.05

Table 2 presents an overall comparison using only AM, IG, and MP as auxiliary tasks. Compared to fine-tuning-based methods ($\mathop {\texttt{FT}}\limits$ and $\mathop {\texttt{GTOT}}\limits$), our proposed methods $\mathop {\texttt{RCGrad}}\limits$ and $\mathop {\texttt{BLO}\text {+}\texttt{RCGrad}}\limits$ demonstrate better performance across 6 out of 8 datasets. Specifically, compared to $\mathop {\texttt{GTOT}}\limits$, $\mathop {\texttt{RCGrad}}\limits$ achieves significant improvements of 2.6% and 5.4% in BACE and BBBP, respectively. Furthermore, $\mathop {\texttt{RCGrad}}\limits$ and $\mathop {\texttt{BLO}\text {+}\texttt{RCGrad}}\limits$ exhibit better performance than $\mathop {\texttt{GS}}\limits$ baselines with significantly improved ROC-AUC of as much as 9.9% in ClinTox. Overall, our proposed methods demonstrate significantly improved performance in smaller datasets compared to fine-tuning and $\mathop {\texttt{GS}}\limits$ baselines. Such consistently superior performance underscores the robustness of our methods, particularly in settings where data is limited and the alignment of gradients is crucial.

In contrast with the previous setup, $\mathop {\texttt{GS}}\limits$ baselines such as $\mathop {\texttt{GCS}}\limits$ and $\mathop {\texttt{GNS}}\limits$ exhibit better performance across almost all datasets. This implies that these methods can be more effective with fewer conflicting tasks, and may struggle to handle a large number of conflicting tasks (Table 1). Similarly, with fewer tasks in this setup, $\mathop {\texttt{MTL}}\limits$ exhibits improved performance compared to the previous setup, thereby indicating diminished negative transfer. This suggests that a smaller and more focused set of auxiliary tasks can lead to more efficient and less conflicting learning dynamics. However, $\mathop {\texttt{PCGrad}}\limits$, $\mathop {\texttt{RCGrad}}\limits$, and $\mathop {\texttt{BLO}\text {+}\texttt{RCGrad}}\limits$, which partially utilize conflicting gradients, show mixed responses to the reduction in the number of auxiliary tasks in this setup. Specifically, $\mathop {\texttt{RCGrad}}\limits$ demonstrates improved performance in smaller datasets (except ClinTox) but a slight decrease in performance in larger datasets, compared to their performance in the previous setup. This can be attributed to the reduced diversity in learning signals provided by a smaller set of auxiliary tasks.

### Comparison using $\mathop {\texttt{Sup}}\limits$ as the pretrained GNN

**Table 3:** Test ROC-AUC using ${\mathop {\mathcal {T}_{a}}\limits }$={AM,CP,EP,IG,MP} and $\mathop {\texttt{Sup}}\limits$

Method	SIDER	ClinTox	BACE	BBBP	Tox21	ToxCast	HIV	MUV
$\mathop {\texttt{FT}}\limits$	61.85 (0.68)	54.16 (5.25)	75.76 (0.65)	66.34 (0.82)	75.64 (0.22)	63.52 (0.23)	72.84 (0.85)	**80.46** (0.19)
$\mathop {\texttt{GTOT}}\limits$	**62.38** (0.39)	55.64 (7.49)	75.82 (2.10)	66.26 (1.87)	75.25 (1.11)	64.00 (0.55)	74.93 (1.50)	**80.42** (0.42)
$\mathop {\texttt{MTL}}\limits$	55.18 (0.96)	47.33 (1.84)	64.84 (2.43)	63.62 (1.08)	73.15 (0.44)	62.06 (2.00)	63.25 (5.15)	69.21 (8.51)
$\mathop {\texttt{GCS}}\limits$	58.39 (0.59)	50.05 (1.48)	74.59 (0.61)	66.67 (2.41)	74.36 (0.43)	63.94 (0.35)	72.23 (0.24)	62.99 (5.35)
$\mathop {\texttt{GNS}}\limits$	60.57 (2.04)	53.52 (5.44)	76.69 (0.88)	68.67 (0.42)	75.37 (0.34)	63.49 (0.12)	74.41 (0.19)	79.72 (0.17)
$\mathop {\texttt{PCGrad}}\limits$	59.83 (0.53)	53.07 (5.12)	71.17 (6.65)	67.18 (1.12)	74.26 (0.53)	63.95 (0.42)	71.80 (0.45)	79.31 (0.74)
$\mathop {\texttt{BLO}}\limits$	60.65 (2.66)	56.10 (4.77)	75.11 (1.19)	67.81 (1.09)^a^	74.57 (0.59)	**64.20** (0.44)	75.05 (0.74)^b^	78.12 (0.68)
$\mathop {\texttt{RCGrad}}\limits$	61.38 (0.74)	**57.36** (3.75)	**77.00** (1.03)	**68.73** (0.76)^a^	**75.67** (0.49)	63.91 (0.23)	**75.60** (0.26)^b^	79.37 (1.74)
$\mathop {\texttt{BLO}\text {+}\texttt{RCGrad}}\limits$	**62.41** (0.81)	**59.45** (3.33)^b^	**77.47 **(0.79)	**69.45** (0.70)^a^	**76.08 **(0.34)^b^	**64.60** (0.28)^ab^	**75.80 **(0.41)^b^	79.97 (1.11)

Best- and second best-performing models are in **bold** and bold. a and b indicate statistical significance compared to the best baselines based on the Wilcoxon signed rank test with p < 0.05

Table 3 presents an overall comparison of adaptation of $\mathop {\texttt{Sup}}\limits$ as the pretrained GNN using all auxiliary tasks. Similar to our findings in the previous section, $\mathop {\texttt{MTL}}\limits$ again results in worse performance compared to fine-tuning methods, thus indicating negative transfer. On the other hand, our proposed methods, specifically $\mathop {\texttt{RCGrad}}\limits$ and $\mathop {\texttt{BLO}\text {+}\texttt{RCGrad}}\limits$, demonstrate improved performance over fine-tuning and $\mathop {\texttt{GS}}\limits$ baselines. Notably, compared to the best fine-tuning baseline $\mathop {\texttt{GTOT}}\limits$, $\mathop {\texttt{BLO}\text {+}\texttt{RCGrad}}\limits$ improved ROC-AUC by 6.8%, 2.2%, and 4.8% in ClinTox, BACE, and BBBP, respectively. Similarly, compared to the best $\mathop {\texttt{GS}}\limits$ baseline $\mathop {\texttt{GNS}}\limits$, $\mathop {\texttt{BLO}\text {+}\texttt{RCGrad}}\limits$ demonstrates notable improvement of 3.0%, 11.1%, and 1.0% in SIDER, ClinTox, and BACE, respectively. Furthermore, compared to $\mathop {\texttt{BLO}}\limits$, which does not explicitly handle conflicting task gradients, $\mathop {\texttt{BLO}\text {+}\texttt{RCGrad}}\limits$ yields consistent improvement across most datasets. Such consistently superior performance of $\mathop {\texttt{BLO}\text {+}\texttt{RCGrad}}\limits$ implies that aligning and extracting informative components out of conflicting task gradients is crucial to improve the generalizablity of pretrained GNNs, regardless of the specific pretraining objective.

Following the similar setup of ${\mathop {\mathtt {Sup\text {-}CP}}\limits }$experiments with a selected subset of auxiliary tasks, Table 5 in Appendix C presents an overall comparison using $\mathop {\texttt{Sup}}\limits$ as the pretrained GNN. Compared to the previous setup with all auxiliary tasks, almost all $\mathop {\texttt{GS}}\limits$ baselines and our proposed method $\mathop {\texttt{RCGrad}}\limits$ exhibit improved performance with fewer auxiliary tasks. This suggests that using a smaller and relevant set of auxiliary tasks can lead to more efficient adaptation, which holds true across different pretrained GNNs. Furthermore, compared to the best $\mathop {\texttt{GS}}\limits$ baseline, $\mathop {\texttt{GNS}}\limits$, our proposed methods $\mathop {\texttt{RCGrad}}\limits$ and $\mathop {\texttt{BLO}\text {+}\texttt{RCGrad}}\limits$ achieve better or comparable performance, particularly on smaller datasets. Additionally, $\mathop {\texttt{BLO}\text {+}\texttt{RCGrad}}\limits$ exhibits significant improvement over $\mathop {\texttt{GCS}}\limits$ in Tox21 and ToxCast.

However, it’s worth noting that when using $\mathop {\texttt{Sup}}\limits$ as the pretrained GNN, all methods, including $\mathop {\texttt{RCGrad}}\limits$ and $\mathop {\texttt{BLO}\text {+}\texttt{RCGrad}}\limits$, yield slightly worse performance compared to when ${\mathop {\mathtt {Sup\text {-}CP}}\limits }$is used as the pretrained GNN. This observation suggests that the $\mathop {\texttt{Sup}}\limits$ pretrained GNN might not capture contextual chemical relationships as effectively as ${\mathop {\mathtt {Sup\text {-}CP}}\limits }$, which was pretrained additionally on the context prediction task. This subtle difference in performance indicates that the choice of pretrained GNN can have an impact on the overall adaptation process. Additional results are presented in Appendix C.

## Conclusion and future work

In this study, we explored multiple adaptation strategies to improve the performance of pretrained GNNs on downstream molecular property prediction tasks. To address the poor generalization performance to such diverse downstream tasks, we introduced two novel methods, $\mathop {\texttt{RCGrad}}\limits$ and $\mathop {\texttt{BLO}\text {+}\texttt{RCGrad}}\limits$, that learn to align conflicting task gradients. Our experiments demonstrate that our proposed methods consistently outperform all fine-tuning and gradient surgery-based approaches, especially on smaller datasets (except ClinTox). This suggests that the adaptation of pretrained GNNs can be a promising direction to boost target task performance, especially with limited labeled data. Our study serves as the first step in exploring the adaptation of pretrained GNNs in molecular property prediction. In future work, we will explore other adaptation strategies to alleviate noisy gradients and to improve task selection with sparser task weights. We will further investigate the benefit of adapting GNNs to diverse downstream molecular regression tasks.

## Figures and Tables

**Algorithm 1:**
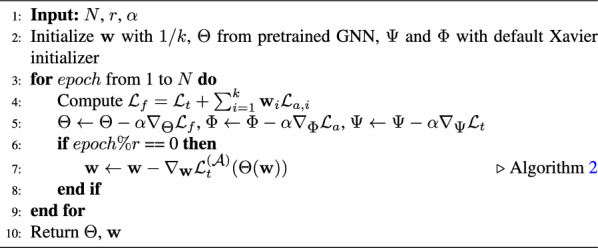
Learning task weights with $\mathop {\texttt{BLO}}\limits$

**Algorithm 2:**
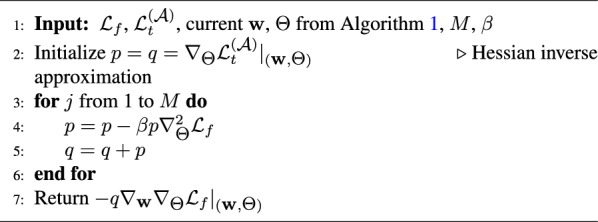
Computing $$\mathop {\nabla _{{\textbf{w}}}}\limits \mathop {\mathcal {L}_{t}^{{(\mathop {\mathcal {A}}\limits )}}}\limits (\Theta (\textbf{w}))$$

**Fig. 5:**
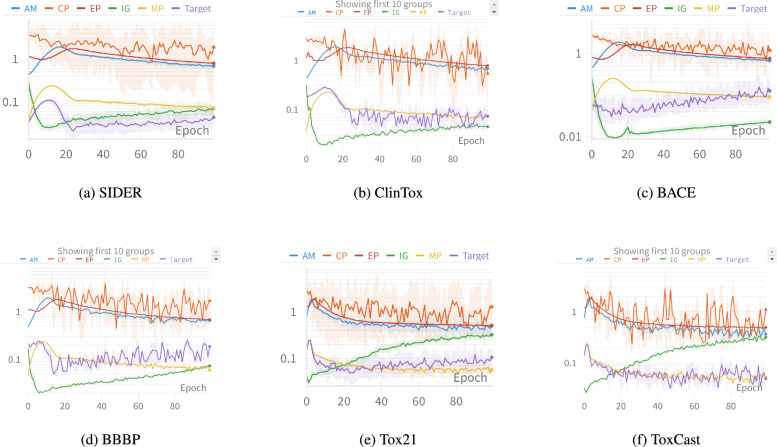
Large variations of scales among task gradients observed across multiple tasks

**Fig. 6:**
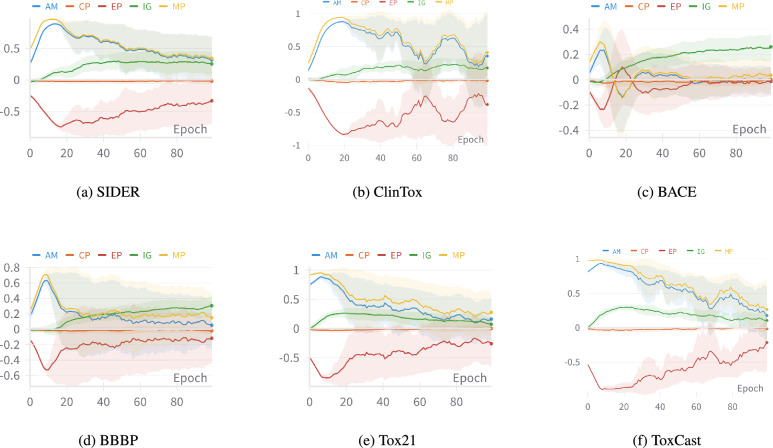
Target task gradient conflicts with EP and CP tasks. ${\mathop {\mathtt {Sup\text {-}CP}}\limits }$is adapted with all auxiliary tasks in a $\mathop {\texttt{MTL}}\limits$ setting

## Data Availability

The data used in the manuscript are publicly and freely available from the MoleculeNet benchmark at https://moleculenet.org/datasets-1. All the code, materials, and instructions on data collection and code execution are publicly available at https://github.com/vishaldeyiiest/GraphTA. All the required softwares and materials to execute the code are public and freely available.

## Appendix A Details on $\mathop {\texttt{BLO}}\limits$

The pseudocode for $\mathop {\texttt{BLO}}\limits$ is provided below:

Notably, the pseudocode for $\mathop {\texttt{BLO}\text {+}\texttt{RCGrad}}\limits$ is very similar to that of $\mathop {\texttt{BLO}}\limits$, except that the gradients ${\textbf{g}}$ for BLO (in Step 5 of Algorithm 1) is computed as ${\textbf{g}} = \mathop {\nabla _{{\Theta }}}\limits \mathop {\mathcal {L}_{f}}\limits$, while ${\textbf{g}}$ for $\mathop {\texttt{BLO}\text {+}\texttt{RCGrad}}\limits$ is computed as described above.

## Appendix B experimental details

### B.1 On auxiliary tasks

We describe the auxiliary tasks and share key insights behind using them:

- Masked Atom Prediction (AM): AM [17] involves predicting the identity of masked atoms within a molecular graph. It helps the GNN to learn the local chemical context and relationships between atoms and bonds, which are crucial for understanding molecular structure and function. The embedding out of GNN is fed to a linear classifier to predict the atom type of masked atoms.
- Edge Prediction (EP): EP [14] focuses on predicting the presence or absence of bonds (edges) between pairs of atoms in a molecular graph. It helps the GNN to capture essential local structural information, including connectivity and spatial arrangement of atoms within molecules. Following existing design [34], the dot product of node embeddings is used to predict the existence of a bond.
- Context Prediction (CP): CP [17] requires the model to predict neighboring graph structures (context) based on an anchor structure. This aids the GNN in distinguishing molecular contexts, enabling the model to capture subgraph-level information. The setup of Hu et al. [17] is followed to extract and distinguish positive and negative subgraph contexts.
- Graph Infomax (IG): IG [33] maximizes the mutual information between local (node) and global (subgraph) representations. This helps the GNN to capture structural patterns, allowing it to understand how atoms form functional groups and larger molecular substructures. The existing setup [33] is followed to train a discriminator model that distinguishes between node embeddings from the same molecular graph and those from a different graph.
- Motif Prediction (MP): MP [28] focuses on predicting the presence of specific recurring substructures (motifs) within a molecule. It helps the GNN to identify structural motifs indicative of chemical properties or functions. This task is formulated as a multi-label binary classification problem with each of 85 motifs^2^ extracted from RDKIT [1] as labels.

Each of these tasks focuses on different aspects of molecular graphs, such as local connectivity, spatial arrangement, contextual information, hierarchical organization, and recurring structural patterns. In essence, these tasks are designed to equip the model with a richer understanding of molecular structures, ultimately improving its ability to generalize and make accurate predictions. Note that designing auxiliary tasks is beyond the scope of this study.

### B.2 Dataset overview

We perform our adaptation experiments on 8 benchmark classification datasets from MoleculeNet [40]. In this section, we give a brief overview and provide preliminary statistics of these datasets.

- BBBP: measures whether a molecule permeates the blood-brain barrier.
- BACE: measures whether a molecule inhibit the $\beta$-secretase 1 (BACE-1) enzyme.
- ClinTox: contains toxicity labels for clinical drugs, facilitating the assessment of drug safety profiles across various targets. It is important to note that these labels reflect both FDA approval outcomes and clinical trial failures due to toxicity. Such outcomes are determined by not just the molecular structures of the drugs. but also by external factors such as genetic predispositions, evaluation methodologies, and environmental conditions. This complexity can make methodological comparisons challenging.
- HIV: measures whether a molecule can prevent antiviral activity against the HIV virus.
- MUV: compiled and refined from PubChem bioassays, evaluating compound activity across multiple targets.
- Tox21: measures toxicity across a range of biological pathways used in the 2014 Tox21 challenge.
- ToxCast: measures compound toxicity across a range of biological systems.

**Table 4:** Overview of benchmark molecular property prediction datasets

Dataset	BBBP	Tox21	ToxCast	SIDER	ClinTox	MUV	HIV	BACE
No. mols	2,039	7.831	8,575	1,427	1,478	93,087	41,127	1,513
No. tasks	1	12	617	27	2	17	1	1
Avg. atoms	24.06	18.57	18.78	33.64	26.16	24.23	25.51	34.09
Avg. diameter	11.32	9.62	9.49	14.14	12.39	12.79	11.98	15.22

### B.3 Additional figures

Figure 5 demonstrates the varying scales of auxiliary task gradient magnitudes when ${\mathop {\mathtt {Sup\text {-}CP}}\limits }$is adapted using all auxiliary tasks in a $\mathop {\texttt{MTL}}\limits$ setting across all datasets. This indicates the need to adjust the gradient norms as proposed in $\mathop {\texttt{GNS}}\limits$ and $\mathop {\texttt{RCGrad}}\limits$. This prevents some auxiliary tasks to dominate over target tasks.

Figure 6 demonstrates that target task gradient conflicts with that of EP and CP tasks across all datasets. This motivates our experimental comparison of all adaptation strategies using a smaller set of more relevant auxiliary tasks.

## Appendix C Additional experimental results

**Table 5:** Test ROC-AUC using ${\mathop {\mathcal {T}_{a}}\limits }$={AM,IG,MP} and $\mathop {\texttt{Sup}}\limits$

Method	SIDER	ClinTox	BACE	BBBP	Tox21	ToxCast	HIV	MUV
$\mathop {\texttt{FT}}\limits$	61.85 (0.68)	54.16 (5.25)	75.76 (0.65)	66.34 (0.82)	**75.64** (0.22)	63.52 (0.23)	72.84 (0.85)	**80.46 **(0.19)
$\mathop {\texttt{GTOT}}\limits$	62.38 (0.39)	55.64 (7.49)	75.82 (2.10)	66.26 (1.87)	75.25 (1.11)	64.00 (0.55)	74.93 (1.50)	**80.42** (0.42)
$\mathop {\texttt{MTL}}\limits$	56.24 (2.79)	53.25 (2.60)	75.92 (1.06)	68.72 (0.73)	72.22 (0.62)	62.94 (0.32)	71.84 (0.95)	74.81 (0.48)
$\mathop {\texttt{GCS}}\limits$	61.31 (0.65)	50.22 (1.60)	75.54 (1.12)	65.23 (1.89)	75.01 (0.30)	64.45 (0.27)	74.03 (0.52)	75.20 (1.99)
$\mathop {\texttt{GNS}}\limits$	**62.47** (0.49)	55.08 (4.51)	**77.28** (1.38)	69.55 (1.10)	74.95 (0.41)	63.94 (0.23)	74.13 (0.34)	77.05 (1.53)
$\mathop {\texttt{PCGrad}}\limits$	57.66 (2.41)	52.20 (3.22)	76.55 (0.96)	69.11 (0.62)	73.01 (0.90)	63.59 (0.48)	71.88 (1.15)	75.28 (1.32)
$\mathop {\texttt{BLO}}\limits$	61.70 (0.86)	56.79 (3.67)	75.25 (1.54)	68.00 (0.88)*	74.53 (0.33)	64.44 (0.73)	75.15 (0.35)^b^	76.97 (3.12)
$\mathop {\texttt{RCGrad}}\limits$	62.10 (1.04)	**58.64** (1.66)^b^	**77.64** (0.80)	**69.63** (0.84)^a^	75.08 (0.53)	**65.09** (0.37)^ab^	**75.63** (0.24)^b^	78.08 (2.78)
$\mathop {\texttt{BLO}\text {+}\texttt{RCGrad}}\limits$	**62.55** (0.85)	**59.31** (3.59)^b^	77.22 (1.53)	**69.67** (0.93)^a^	**75.74** (0.58)^b^	**65.18** (0.44)^ab^	**75.78** (0.21)^b^	**78.37** (2.31)

Best- and second best-performing models are in **bold** and bold. a and b indicate statistical significance compared to the best baselines based on the Wilcoxon signed rank test with $p < 0.05$

Table 5 presents an overall comparison when $\mathop {\texttt{Sup}}\limits$ is adapted using only AM, IG, and MP as auxiliary tasks. Compared to fine-tuning-based methods ($\mathop {\texttt{FT}}\limits$ and $\mathop {\texttt{GTOT}}\limits$), our proposed methods $\mathop {\texttt{RCGrad}}\limits$ and $\mathop {\texttt{BLO}\text {+}\texttt{RCGrad}}\limits$ demonstrate better performance across 7 out of 8 datasets. Specifically, compared to $\mathop {\texttt{GTOT}}\limits$, both $\mathop {\texttt{RCGrad}}\limits$ and $\mathop {\texttt{BLO}\text {+}\texttt{RCGrad}}\limits$ achieve significant improvements of up to 5.1% and 1.8% in BBBP and ToxCast, respectively. Furthermore, $\mathop {\texttt{RCGrad}}\limits$ and $\mathop {\texttt{BLO}\text {+}\texttt{RCGrad}}\limits$ exhibit better performance than $\mathop {\texttt{GS}}\limits$ baselines with significantly improved ROC-AUC of as much as 7.7% and 2.2% in ClinTox and HIV, respectively. Overall, both $\mathop {\texttt{RCGrad}}\limits$ and $\mathop {\texttt{BLO}\text {+}\texttt{RCGrad}}\limits$ outperform fine-tuning methods, while achieving competitive or better performance than $\mathop {\texttt{GS}}\limits$ baselines across all datasets. Such consistently superior performance across multiple setups and pretrained GNNs underscores the robustness of our methods.

## Footnotes

1 https://github.com/snap-stanford/pretrain-gnns
